# Serum C-reactive protein and procalcitonin values in acute Q fever, scrub typhus, and murine typhus

**DOI:** 10.1186/s12879-020-05058-8

**Published:** 2020-05-12

**Authors:** I-Fan Lin, Jiun-Nong Lin, Chia-Ta Tsai, Yu-Ying Wu, Yen-Hsu Chen, Chung-Hsu Lai

**Affiliations:** 1 Division of Infectious Diseases, Department of Internal Medicine, E-Da Hospital, I-Shou University, No.1, Yi-Da Road, Jiao-Su Village, Yan-Chao District, Kaohsiung City, 824 Taiwan; 2 School of Medicine, College of Medicine, I-Shou University, No.1, Yi-Da Road, Jiao-Su Village, Yan-Chao District, Kaohsiung City, 824 Taiwan; 3 Department of Critical Care Medicine, E-Da Hospital, I-Shou University, Kaohsiung City, Taiwan; 4 Department of Infection Control, E-Da Hospital, I-Shou University, Kaohsiung City, 824 Taiwan; 5 Department of Neurosurgery, E-Da Hospital, I-Shou University, Kaohsiung City, 824 Taiwan; 6 Department of Internal Medicine, Kaohsiung Municipal Ta-Tung Hospital, Kaohsung, Taiwan; 7 School of Medicine, Graduate Institute of Medicine, Sepsis Research Center, Center of Dengue Fever Control and Research, Kaohsiung Medical University, Kaohsung, Taiwan; 8 Department of Biological Science and Technology, College of Biological Science and Technology, National Chiao Tung University, Hsinchu, Taiwan

**Keywords:** C-reactive protein, Procalcitonin, Doxycycline, Murine typhus, Q fever, Scrub typhus, Rickettsioses

## Abstract

**Background:**

Although C-reactive protein (CRP) and procalcitonin (PCT) are widely used inflammatory markers for infectious diseases, their role and potential application for rickettsioses were rarely studied.

**Methods:**

A retrospective chart review and serological study were conducted in patients with rickettsioses. The clinical presentations, characteristics, laboratory data, and treatment responses were recorded and their associations with CRP and PCT values were analyzed.

**Results:**

A total of 189 cases of rickettsioses, including 115 cases of acute Q fever (60.8%), 55 cases of scrub typhus (29.1%), and 19 cases of murine typhus (10.1%) were investigated. Both CRP and PCT values increased in the acute phase and declined in the convalescent phase. In the acute phase, mean CRP and PCT values were 78.2 ± 63.7 mg/L and 1.05 ± 1.40 ng/mL, respectively. Percentages of patients falling under different cut-off values of CRP and PCT were calculated systematically. Only 10.8% of CRP was > 150 mg/L and 14.2% of PCT was > 2.0 ng/mL. Patients with delayed responses to doxycycline treatment (> 3 days from treatment to defervescence) had significantly higher CRP values (102.7 ± 77.1 vs. 72.2 ± 58.2 mg/L, *p* = 0.041) and more PCT > 1.0 ng/ml (48.4% vs. 26.0%, *p* = 0.019) in the acute phase; higher CRP values (19.1 ± 37.4 vs. 3.6 ± 13.1 mg/L, *p* = 0.049) and more PCT > 0.5 ng/ml (19.2% vs. 1.4%, *p* = 0.005) in the convalescent phase. Correlation analysis was conducted for patients with acute Q fever. CRP and PCT values were positively correlated to each other, and both markers also had a positive correlation with serum aspartate transaminase values. Both CRP and PCT values and white blood cell counts were positively correlated to the days needed from doxycycline treatment to defervescence.

**Conclusion:**

CRP and PCT values might be useful in clinical investigations for patients with suspected rickettsioses and in predicting the response to doxycycline treatment for rickettsioses.

## Background

Rickettsioses consist of a diverse group of zoonotic infections, including rickettsial diseases, ehrlichiosis, anaplasmosis, scrub typhus (caused by *Orientia tsutsugamushi*), and Q fever (caused by *Coxiella burnetii*) [1]. They typically manifest as febrile illness with headache and rashes, and elevation in transaminases, and would be listed in the differential diagnoses of fever in endemic areas [2–4]. Because of the character of intracellular survival which need special methods for culture of the causative pathogens, identification and isolation of these microorganisms are clinically challenging. The definite diagnosis of rickettsioses often counts on molecular method with polymerase chain reaction (PCR) for deoxyribonucleic acid (DNA) detection in specimen and serological assays with immunofluorescent assay (IFA) for detection of specific antibodies against to the causative microorganisms. However, these diagnostic tools are not universally available in clinical practice and medical care settings.

C-reactive protein (CRP) is an acute phase protein and the production of CRP is stimulated by a proinflammatory cytokine, interleukin 6 (IL-6) [5], involving in the host defense against bacterial infections [6]. Procalcitonin (PCT), a precursor of calcitonin, was described to be associated with sepsis and infection [7] and was subsequently widely used as a clinical marker of sepsis [8]. It has a higher diagnostic accuracy than CRP to differentiate bacterial infections from noninfectious causes and viral infections in patients hospitalized for suspected infectious diseases [9]. It was intuitive and practical to use CRP and PCT values during initial evaluation of patients with acute febrile illnesses. However, there was no systemic investigation on CRP and PCT values in rickettsioses yet. In addition, the trend of CRP and PCT values, their correlation with other clinical biomarkers, and whether CRP and PCT values could be predictors of treatment response in rickettsioses were not well understood.

Geographic distributions of rickettsial diseases varied, such as Rocky mountain spotted fever in the North America [10] and murine typhus in tropical and subtropical areas, including the Mediterranean [11]. According to previous multi-center and nationwide database studies, acute Q fever, scrub typhus, and murine typhus comprised the three most common rickettsioses in Taiwan [12, 13] and we focused on these diseases. In clinical practice, these three diseases could be possibly differentiated by different environmental exposure, animal contact history, and clinical manifestations, but these information recalled by the patients might be unreliable. Sometimes rickettsioses could even manifest with atypical presentations [11]. For laboratory data, hepatitis and thrombocytopenia were widely mentioned as the characteristics of rickettsioses [4, 14–20]. Elevation of CRP was found in some studies of rickettsioses, but the values of PCT were rarely been mentioned [18, 20–25].

The aim of this study was to investigate the changes of CRP and PCT values in acute and convalescent phases of rickettsioses and the association of these two markers with other biochemistry markers and with the response to doxycycline treatment in patients with rickettsioses.

## Methods

### Clinical cases of rickettsioses

This was a retrospective study of rickettsioses conducted in E-Da Hospital, Kaohsiung City, Taiwan, from April 01, 2004 to August 31, 2010. This study was approved by the Ethics Committee of the E-Da Hospital (EMRP-097-117). The committee waived the need for written informed consent because the demographic information and clinical data were retrospectively recorded, and all data were collected anonymously. Chart review of patients with rickettsioses confirmed by the Centers for Disease Control, Taiwan (Taiwan CDC) was performed. Clinical presentations, patient characteristics, results of laboratory examinations, and treatment responses were collected. A delayed response to treatment was defined as more than 3 days from administration of doxycycline to defervescence. Patients who received fluoroquinolones or achieved defervescence before the administration of doxycycline were excluded for analysis of response to treatment.

### Diagnosis of acute Q fever, scrub typhus, and murine typhus

Q fever, scrub typhus, and murine typhus are notifiable diseases in Taiwan and reporting suspected cases to Taiwan CDC is mandatory for clinicians. The paired blood specimens (obtained in acute and convalescent phases) were sent to the contracted laboratories of Taiwan CDC for confirmation tests. The diagnosis could be made either by serologic tests using indirect IFA that detects specific antibodies or by PCR that detects the DNA of causative pathogens in the blood. The indirect IFA targeted specific antibodies to *C. burnetii*, *O. tsutsugamushi*, and *Rickettsia typhi* for diagnosis of Q fever, scrub typhus, and murine typhus respectively, as previously prescribed^13^. Acute Q fever was diagnosed by either an anti-phase II antigen IgM titer of > 80 or a > 4-fold rise of anti-phase II antigen immunoglobulin G (IgG) titer in paired serum samples. Scrub typhus was confirmed by either an immunoglobulin M (IgM) titer > 80 or a > 4-fold rise in IgG titer in paired sera for Karp, Kato, and Gilliam strains of *O. tsutsugamushi*. Murine typhus was diagnosed by an IgM titer > 80 or a > 4-fold rise in IgG titer against to *R. typhi* in paired sera [15].

### Measurement of serum C-reactive protein and serum procalcitonin

The clinical CRP values were retrospectively collected from clinical chart review and they were measured by Nephelometry method (Dade Behring BN II nephelometer [Dade Behring, Siemens]). Missing data of CRP was measured by human CRP enzyme-linked immunosorbent assay (ELISA) Kit (ANOGEN, Yes Biotech Laboratories Ltd., Canada) if serum was available. The serum PCT values were measured by IDAS® BRAHMS PCT test. The molecular structure of PCT is stable under room temperature and − 80 °C, and the freeze-thaw effect does not affect measured PCT values [26]. Sampling and testing were performed according to manufacturer instruction manuals. Serum was the residual specimen obtained for the diagnosis-related purposes of Q fever, scrub typhus, murine typhus for the Taiwan CDC and it was stored at − 80 °C until analysis.

### Statistical analysis

Continuous variables were analyzed by Student’s t-test and categorical variables by Chi-square or Fisher’s exact test. Pearson correlation coefficient was used in correlation analyses. Two-tailed *p*-values were calculated and a *p*-value of less than 0.05 was regarded as statistically significant. SPSS software for Windows (Release 15.0; SPSS, Chicago, IL) was used for statistical analysis.

## Results

From April 01, 2004 to August 31, 2010, a total of 189 cases of rickettsioses confirmed by Taiwan CDC in E-Da hospital were included. Among them, 115 cases were acute Q fever (60.8%), 55 cases were scrub typhus (29.1%), and 19 cases were murine typhus (10.1%). The patients’ demographic data were shown in Table 1. The patients in all three diseases were predominantly male in their 40s, with age ranging from 16 to 80 years old. In average, 77.3% of patients with these rickettsial diseases could achieve defervescence within 3 days of doxycycline treatment after excluding those who had received fluoroquinolones or achieved defervescence before administration of doxycycline. Leukocytosis (white blood cell [WBC] count > 10,000/uL), thrombocytopenia (platelet count < 150,000/uL), and elevation of any liver enzymes (aspartate transaminase [AST] > 38 U/L or alanine transaminase [ALT] > 44 U/L) accounted for 11.1, 71.4, and 96.3%, respectively.

**Table 1:** The demographic data of patients of acute Q fever, scrub typhus and, murine typhus

	Acute Q fever (*n* = 115)	Scrub typhus (*n* = 55)	Murine typhus (*n* = 19)	Total (*n* = 189)
Demographics				
Male	108 (93.9%)	33 (60.0%)	15 (78.9%)	156 (82.5%)
Age (years) (mean ± SD)	44.8 ± 12.1	44.3 ± 16.9	49.8 ± 14.6	45.1 ± 13.9
Admission	93 (80.9%)	52 (94.5%)	18 (94.7%)	163 (86.2%)
Treatment to defervescence ≤3 days ^a^	71/89 (79.8%)	38/50 (76%)	10/15 (66.7%)	119/154 (77.3%)
Underlying conditions				
Old age (≥65 years)	10 (8.7%)	12 (21.8%)	2 (10.5%)	24 (12.7%)
Hypertension	13 (11.3%)	7 (12.7%)	1 (5.3%)	21 (11.1%)
Diabetes mellitus	8 (7.0%)	4 (7.3%)	2 (10.5%)	14 (7.4%)
COPD	2 (1.7%)	0 (0%)	0 (0%)	2 (1.1%)
Cirrhosis	1 (0.9%)	4 (7.3%)	0 (0%)	5 (2.6%)
Alcoholism	10 (8.7%)	3 (5.5%)	1 (5.3%)	14 (7.4%)
Chronic hepatitis B	25/108 (23.1%)	6/46 (13.0%)	2 /18 (11.1%)	33/172 (19.2%)
Chronic hepatitis C	9/108 (8.3%)	3/46 (6.5%)	1/18 (5.6%)	13/172 (7.6%)
Chronic kidney disease	0 (0%)	0 (0%)	0 (0%)	0 (0%)
Heart failure	2 (1.7%)	1 (1.8%)	0 (0%)	3 (1.6%)
Old stroke	1 (0.9%)	0 (0%)	0 (0%)	1 (0.5%)
Hematologic malignancy	1 (0.9%)	0 (0%)	0 (0%)	1 (0.5%)
Solid tumor	1 (0.9%)	0 (0%)	0 (0%)	1 (0.5%)
Connective tissue disease	0 (0%)	1 (1.8%)	0 (0%)	1 (0.5%)
Laboratory data (mean ± SD)				
WBC (/uL)	6153 ± 2564	7650 ± 2657	8758 ± 6837	6850 ± 3368
Leukocytosis (WBC > 10,000/uL)	5/115 (4.3%)	10/55 (18.2%)	6/19 (31.6%)	21/189 (11.1%)
Hemoglobin (g/dL)	14.6 ± 1.3	12.9 ± 1.7	14.0 ± 2.1	14.0 ± 1.7
Anemia (Hb < 10 g/dL)	0/115 (0%)	3/55 (5.5%)	0/19 (0%)	3/189 (1.6%)
Platelet (1000/uL)	139 ± 57	137 ± 69	148 ± 79	139 ± 63
Thrombocytopenia (< 150,000/uL)	85/115 (73.9%)	39/55 (70.9%)	11/19 (57.9%)	135/189 (71.4%)
AST (U/L)	118 ± 81	165 ± 282	162 ± 123	136 ± 169
AST > 38 U/L	109/111 (98.2%)	47/53 (88.7%)	19/19 (100%)	175/183 (95.6%)
ALT (U/L)	129 ± 79	149 ± 198	249 ± 440	147 ± 184
ALT> 44 U/L	111/114 (97.4%)	42/53 (79.2%)	18/18 (100%)	171/185 (92.4%)

*SD* standard deviation, *COPD* chronic obstructive pulmonary disease, *TB* tuberculosis, *WBC* white blood cell, *AST* aspartate transaminase, *ALT* alanine transaminase

^a^Patients who received fluoroquinolones or achieved defervescence before intervention of doxycycline treatment were excluded

**Fig. 1:**
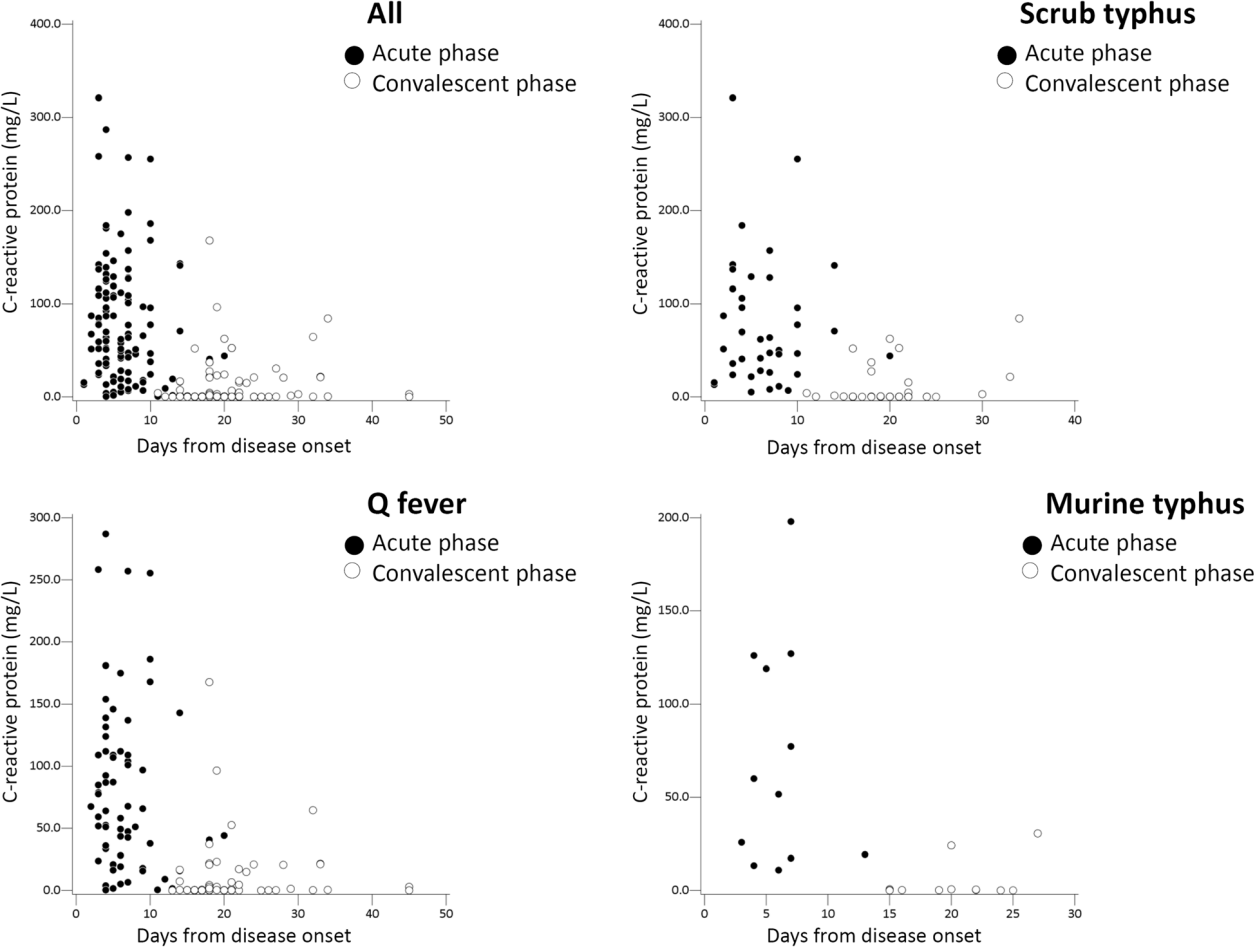
C-reactive protein (CRP) values and days from disease onset of acute Q fever, scrub typhus, and murine typhus

**Fig. 2:**
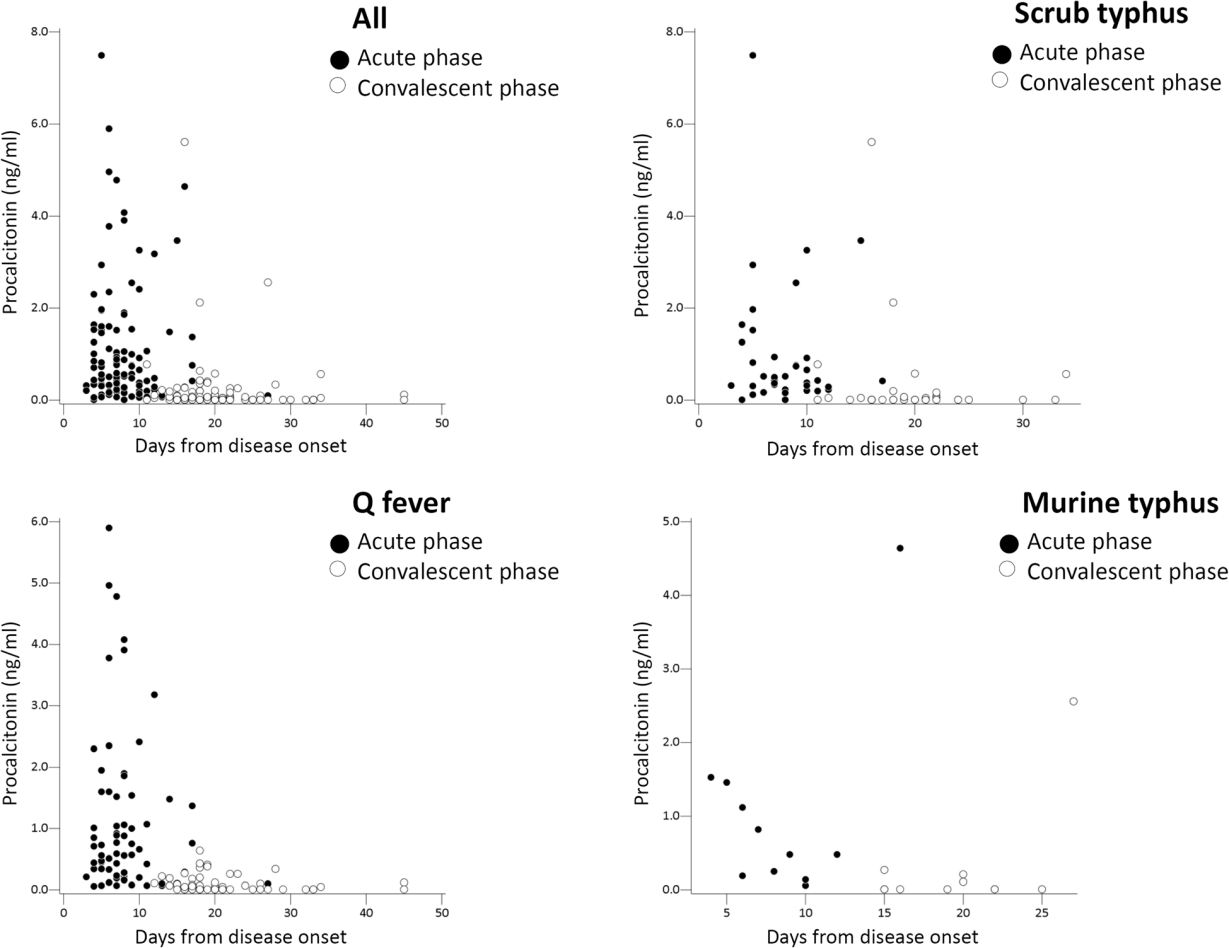
Procalcitonin (PCT) values and days from disease onset of acute Q fever, scrub typhus, and murine typhus

The relation between days from disease onset of the three rickettsial diseases and CRP or PCT values was shown in Figs. 1 and 2, respectively. Tests for the convalescent phase were performed 11–45 days from the disease onset (average, 20.2 ± 5.5 days; median, 19 days). In all three rickettsioses, both CRP and PCT values were increased in the acute phase and decreased in the convalescent phase. CRP and PCT values in the acute and convalescent phases of acute Q fever, scrub typhus, and murine typhus were listed in Table 2. There was no difference in mean CRP and PCT values of both acute and convalescent phases between the three rickettsioses. Subgrouping the CRP and PCT values in different cut-off values was conducted for possible clinical applications. Only 10.8% of patients had CRP levels > 150 mg/L and only 14.2% of patients had PCT levels > 2.0 ng/mL in the acute phase of three rickettsioses.

**Table 2:** C-reactive protein (CRP) and procalcitonin (PCT) values in the acute and convalescent phases of acute Q fever, scrub typhus, and murine typhus

		Acute Q fever (*n* = 115)	Scrub typhus (*n* = 55)	Murine typhus (*n* = 19)	All (*n* = 189)	*p*
CRP (mg/L)						
Acute phase	Mean ± SD	80.6 ± 60.9	74.2 ± 66.8	75.0 ± 74.2	78.2 ± 63.7	0.824
	> 25	78.5% (84/107)	74.5% (38/51)	72.2% (13/18)	76.7% (135/176)	0.766
	> 50	68.2% (73/107)	58.8% (30/51)	50.0% (9/18)	63.6% (112/176)	0.231
	> 75	45.8% (49/107)	37.3% (19/51)	38.9% (7/18)	42.6% (75/176)	0.564
	> 100	33.6% (36/107)	25.5% (13/51)	27.8% (5/18)	30.7% (54/176)	0.560
	> 150	11.2% (12/107)	9.8% (5/51)	11.1% (2/18)	10.8% (19/176)	1.000
	> 200	3.7% (4/107)	3.9% (2/51)	5.6% (1/18)	4.0% (7/176)	0.853
Convalescent phase^**a**^	Mean ± SD	8.9 ± 25.7	7.3 ± 19.2	4.8 ± 10.6	8.0 ± 22.4	0.827
	> 25	4.8% (3/62)	11.1% (4/36)	8.3% (1/12)	7.3% (8/110)	0.476
	> 50	4.8% (3/62)	8.3% (3/36)	0% (0/12)	5.5% (6/110)	0.702
	> 75	3.2% (2/62)	2.8% (1/36)	0% (0/12)	2.7% (3/110)	1.000
	> 100	1.6% (1/62)	0% (0/36)	0% (0/12)	0.9% (1/110)	1.000
PCT (ng/mL)						
Acute phase	Mean ± SD	1.09 ± 1.40	0.99 ± 1.46	0.97 ± 1.22	1.05 ± 1.40	0.904
	> 0.25	75.3% (70/93)	63.3% (31/49)	61.5% (8/13)	70.3% (109/155)	0.254
	> 0.5	54.8% (51/93)	40.8% (20/49)	46.2% (6/13)	49.7% (77/155)	0.273
	> 1.0	31.2% (29/93)	24.5% (12/49)	38.5% (5/13)	29.7% (46/155)	0.545
	> 1.5	21.5% (20/93)	22.4% (11/49)	15.4% (2/13)	21.3% (33/155)	0.955
	> 2.0	14.0% (13/93)	16.3% (8/49)	7.7% (1/13)	14.2% (22/155)	0.825
	> 4.0	6.5% (6/93)	4.1% (2/49)	7.7% (1/13)	5.8% (9/155)	0.764
Convalescent phase^**a**^	Mean ± SD	0.09 ± 0.13	0.29 ± 0.98	0.27 ± 0.73	0.17 ± 0.60	0.209
	> 0.25	14.3% (10/70)	13.5% (5/37)	16.7% (2/12)	14.3% (17/119)	1.000
	> 0.5	1.4% (1/70)	13.5% (5/37)	8.3% (1/12)	5.9% (7/119)	0.030
	> 1.0	0% (0/70)	5.4% (2/37)	8.3% (1/12)	2.5% (3/119)	0.084
	> 1.5	0% (0/70)	5.4% (2/37)	8.3% (1/12)	2.5% (3/119)	0.084
	> 2.0	0% (0/70)	5.4% (2/37)	8.3% (1/12)	2.5% (3/119)	0.084

*SD* standard deviation

^a^Tests for the convalescent phase were performed 11–45 days from the disease onset (average, 20.2 ± 5.5 days; median, 19 days)

CRP and PCT values in all patients with and without delayed treatment responses to doxycycline were listed in Table 3. Among them, six patients had received macrolides (azithromycin or clarithromycin) before initiation of doxycycline. Only one of the six patients completed the three-day azithromycin treatment (Q fever), and the other five patients received doxycycline after only 1 day of azithromycin (one case of Q fever and four cases of scrub typhus). Because of the incomplete treatments of macrolides, these patients were not excluded from the analysis of treatment response to doxycycline.

**Table 3:** C-reactive protein (CRP) and procalcitonin (PCT) value in patients of acute Q fever, scrub typhus, and murine typhus with and without delayed responses to doxycycline treatment^a^

		Treatment to defervescence ≤3 days	Treatment to defervescence > 3 days	*p*
CRP (mg/L)				
Acute phase (Mean ± SD)		72.2 ± 58.2	102.7 ± 77.1	0.041*
	> 25	75.9% (85/112)	84.8% (28/33)	0.276
	> 50	61.6% (69/112)	75.8% (25/33)	0.135
	> 75	40.2% (45/112)	51.5% (17/33)	0.247
	> 100	26.8% (30/112)	45.5% (15/33)	0.042*
	> 150	8.0% (9/112)	21.2% (7/33)	0.053
	> 200	2.7% (3/112)	9.1% (3/33)	0.131
Convalescent phase^**b**^ (Mean ± SD)		3.6 ± 13.1	19.1 ± 37.4	0.049*
	> 25	3.2% (2/62)	15.4% (4/26)	0.060
	> 50	1.6% (1/62)	15.4% (4/26)	0.025*
	> 75	1.6% (1/62)	7.7% (2/26)	0.207
	> 100	0% (0/62)	3.8% (1/26)	0.295
PCT (ng/mL)				
Acute phase (Mean ± SD)		0.98 ± 1.40	1.39 ± 1.56	0.167
	> 0.25	70.0% (70/100)	77.4% (24/31)	0.423
	> 0.5	47.0% (47/100)	58.1% (18/31)	0.282
	> 1.0	26.0% (26/100)	48.4% (15/31)	0.019*
	> 1.5	20.0% (20/10)	29.0% (9/31)	0.290
	> 2.0	12.0% (12/100)	19.4% (6/31)	0.370
	> 4.0	5.0% (5/100)	9.7% (3/31)	0.393
Convalescent phase^**b**^ (Mean ± SD)		0.08 ± 0.26	0.39 ± 1.09	0.166
	> 0.25	6.9% (5/72)	30.8% (8/26)	0.005*
	> 0.5	1.4% (1/72)	19.2% (5/26)	0.005*
	> 1.0	1.4% (1/72)	3.8% (1/26)	0.462
	> 1.5	1.4% (1/72)	3.8% (1/26)	0.462
	> 2.0	1.4% (1/72)	3.8% (1/26)	0.462

*SD* standard deviation

*p < 0.05 indicates statistical significance

^a^Patients who received fluoroquinolones or achieved defervescence before intervention of doxycycline treatment were excluded

^b^Tests for the convalescent phase were performed 11–45 days from the disease onset (average, 20.0 ± 5.6 days; median, 19 days)

Patients with delayed treatment responses had higher mean CRP values than those without delayed responses in both acute (102.7 ± 77.1 vs. 72.2 ± 58.2 mg/L, *p* = 0.041) and convalescent (19.1 ± 37.4 vs. 3.6 ± 13.1 mg/L, *p* = 0.049) phases. Different cut-off values for CRP and PCT were further analyzed between the two groups of treatment response. Percentages of CRP values > 100 mg/L in the acute phase (45.5% vs. 26.8%, *p* = 0.042) and > 50 mg/L in the convalescent phase (15.4% vs. 1.6%, *p* = 0.025) were higher in patient with delayed defervescence. For the PCT value, percentages of PCT > 1.0 ng/mL in the acute phase (48.4% vs. 26.0%, *p* = 0.019) and > 0.5 ng/mL in the convalescent phase (19.2% vs. 1.4%, *p* = 0.005) were significantly higher in the group of delayed defervescence.

**Fig. 3:**
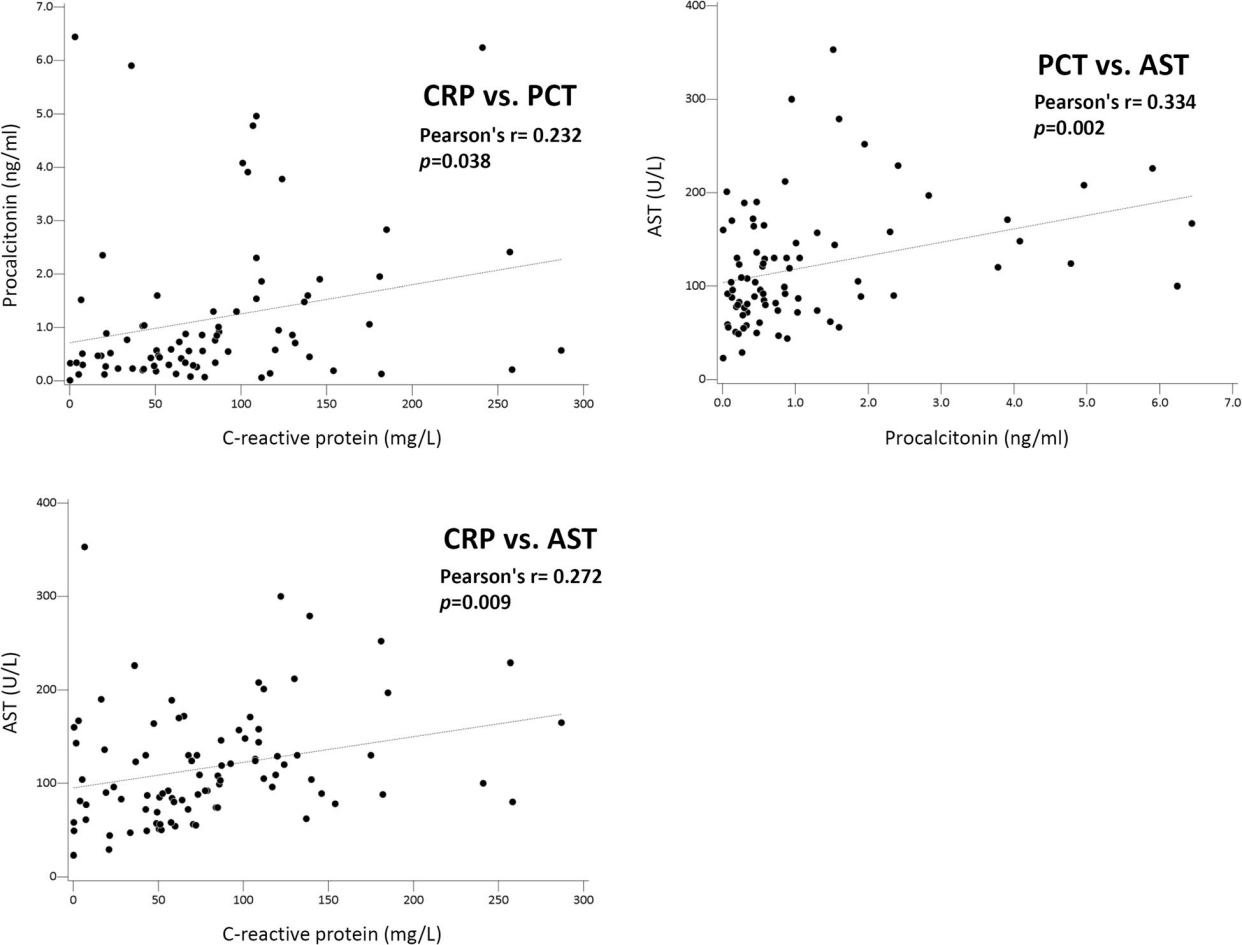
The correlation between C-reactive protein (CRP), procalcitonin (PCT), and aspartate transaminase (AST) values in acute Q fever within 7 days from disease onset

**Fig. 4:**
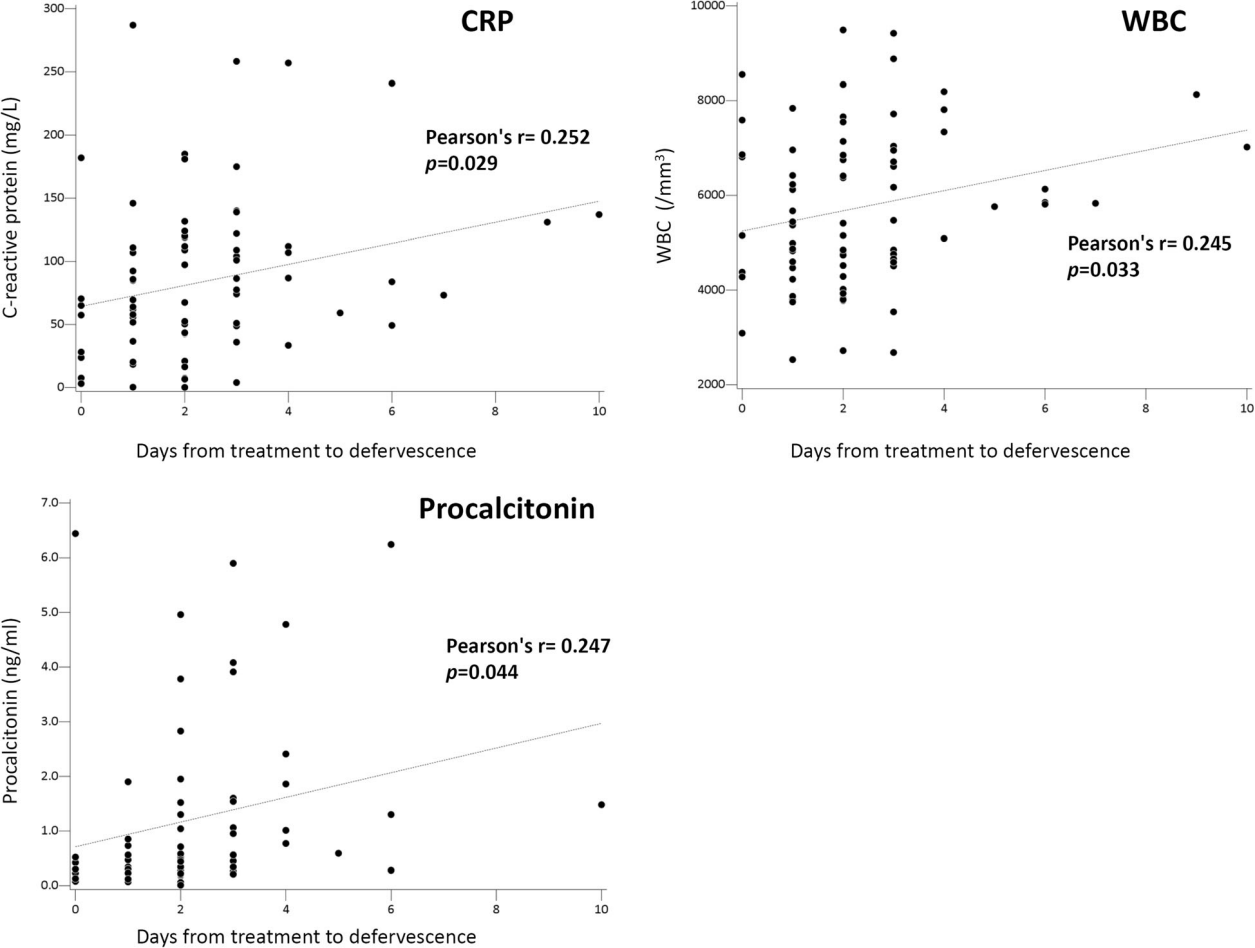
The correlation between values of C-reactive protein (CRP), procalcitonin (PCT), and white blood cell (WBC) count, and the response to doxycycline treatment in acute Q fever. The laboratory tests were done within 7 days from disease onset

In subgroup analysis of acute Q fever patients, those with delayed defervescence had higher CRP and PCT values in both acute and convalescent phase, though not reaching statistical significance. Patients with delayed defervescence also had higher percentage of PCT > 1.0 ng/mL in the acute phase and PCT > 0.25 ng/mL in the convalescent phase (57.1% vs. 29.5%, *p* = 0.050; 33.3% vs. 7.1%, *p* = 0.036) (Table 4). Correlation analysis of CRP, PCT, AST and WBC values with days from doxycycline treatment to defervescence was also conducted in the subgroup analysis of acute Q fever patients; the laboratory data were obtained within 7 days after the disease onset. CRP, PCT, and AST values were significantly correlated with each other (CRP and PCT, *r* = 0.232, *p* = 0.038; CRP and AST, *r* = 0.272, *p* = 0.009; PCT and AST, *r* = 0.334, *p* = 0.002) (Fig. 3). The days from doxycycline treatment to defervescence has significantly positive correlation with CRP, PCT, and WBC counts (CRP, *r* = 0.252, *p* = 0.029; PCT, *r* = 0.247, *p* = 0.044; WBC, *r* = 0.245, *p* = 0.033) (Fig. 4).

**Table 4:** C-reactive protein (CRP) and procalcitonin (PCT) values in patients of acute Q fever with and without delayed responses to doxycycline treatment^a^

		Treatment to defervescence ≤3 days	Treatment to defervescence > 3 days	*p*
CRP (mg/L)				
Acute phase (Mean ± SD)		79.1 ± 61.2	107.8 ± 68.8	0.096
	> 25	77.6% (52/67)	94.1% (16/17)	0.174
	> 50	67.2% (45/67)	82.4% (14/17)	0.373
	> 75	44.8% (30/67)	58.8% (10/17)	0.300
	> 100	34.3% (23/67)	47.1% (8/17)	0.331
	> 150	10.4% (7/67)	17.6% (3/17)	0.415
	> 200	3.0% (2/67)	11.8% (2/17)	0.181
Convalescent phase (Mean ± SD) ^b^		5.3 ± 17.4	20.7 ± 45.2	0.103
	> 25	3.1% (1/32)	7.7% (1/13)	0.499
	> 50	3.1% (1/32)	7.7% (1/13)	0.499
	> 75	3.1% (1/32)	7.7% (1/13)	0.499
	> 100	0% (0/46)	7.1% (1/14)	0.289
PCT (ng/mL)				
Acute phase (Mean ± SD)		1.07 ± 1.45	1.67 ± 1.78	0.187
	> 0.25	75.4% (46/61)	85.7% (12/14)	0.502
	> 0.5	49.2% (30/61)	78.6% (11/14)	0.073
	> 1.0	29.5% (18/61)	57.1% (8/14)	0.050*
	> 1.5	23.0% (14/61)	28.6% (4/14)	0.731
	> 2.0	13.1% (8/61)	21.4% (3/14)	0.420
	> 4.0	6.6% (4/61)	14.3% (2/14)	0.311
Convalescent phase (Mean ± SD)^b^		0.06 ± 0.10	0.17 ± 0.20	0.098
	> 0.25	7.1% (3/42)	33.3% (4/12)	0.036*
	> 0.5	0% (0/42)	8.3% (1/12)	0.222
	> 1.0	0% (0/42)	0% (0/12)	NC

*SD* standard deviation, *NC* not calculated

*p < 0.05 indicates statistical significance

^a^Patients who received fluoroquinolones or achieved defervescence before intervention of doxycycline treatment were excluded

^b^Tests for the convalescent phase of acute Q fever were performed 12–45 days from the disease onset (average, 20.3 ± 6.1 days; median, 18.5 days)

## Discussion

This is a study aimed at the specificity of CRP and PCT values in three rickettsial diseases. Like our previous reports [15, 27, 28], a male predominance was found. Our hospital is in a suburban area and a large portion of people are workers or farmers engaged in field activities, increasing the chance of contracting rickettsioses. Furthermore, Q fever accounts 60.8% of our cases and a sex discrepancy in acute Q fever which might result from the protective effect of female hormones in *C. burnetii* infection had been found in the animal study [29]. We also found relatively high rates of chronic hepatitis B and C. Based on our previous study, viral hepatitis would not affect the results regarding liver biochemistry in rickettsioses [30]. Because of the national health insurance, the accessibility of healthcare facilities, and the affordable medical expenses, patients with acute febrile illness in Taiwan tended to seek medical attention at the emergency department and were often admitted. The condition contributed to the high admission rate in this study.

Previous literature had reported that CRP and PCT values reflected severity of bacterial infections [9, 31]. Our study found that both serum CRP and PCT values rose in the acute phase and declined in the convalescent phase in acute Q fever, scrub typhus, and murine typhus (Figs. 1 and 2). The findings suggested both CRP and PCT values may reflect disease progression in rickettsioses as well as other common bacterial infections.

We found that only 10.8% of patients with rickettsioses had a CRP value > 150 mg/L and only 14.2% of that had a PCT value > 2.0 ng/mL in the acute phase (Table 2). This implied that for febrile patients presenting with CRP values higher than 150 mg/L or PCT values higher than 2.0 ng/mL, the diagnosis of rickettsioses might be less likely; other bacterial infections should be put in the list of differential diagnosis. From a different perspective, Lee CS [32] et al found patients with a PCT value lower than 1.3 ng/mL had a 25.4 times higher risk of having scrub typhus than of having *E. coli* bacteremia. When comparing with dengue fever, Chang K [19] et al found that a CRP value more than 31.9 mg/L was more suggestive of rickettsioses rather than dengue fever, which was also an endemic febrile illness in tropical areas. Because of the readiness and availability in most hospitals, CRP and PCT tests could be useful tools in the early differentiation of endemic febrile illness and commonly encountered bacterial infections.

Our previous study had showed 83.0% (88/106) of patients with acute Q fever, scrub typhus, and murine typhus achieved defervescence within 3 days of doxycycline treatment, and delayed defervescence was associated with patients presenting with jaundice, relative bradycardia, and absence of headache [15]. In present study, around 70% of patients achieved defervescence within 3 days of doxycycline treatment (Table 1) and both CRP and PCT values were related to treatment responses. Patients with delayed responses to doxycycline treatment had significantly higher CRP values and more PCT > 1.0 ng/ml in the acute phase and higher CRP values and more PCT > 0.5 ng/ml in the convalescent phase (Table 3). Several previous studies had shown CRP and PCT values reflected disease severity of rickettsioses [18, 20–24, 33, 34]. Our studies, with linkage of clinical symptoms and signs, CRP and PCT values, could provide another perspective to evaluate and predict treatment response to doxycycline.

In the subgroup analysis of 115 patients with acute Q fever, CRP and PCT values within 7 days of onset of acute Q fever were positively correlated, and both markers were positively correlated with the AST value significantly (Fig. 3), which was not previously reported. The results echoed several previous studies that abnormal liver function was found in the acute phase of acute Q fever [14–19]. However, while nearly all Q fever patients in our study had elevated levels of serum transaminase, a Q fever inpatient cohort in the Netherlands showed only 32.3% (59/183) of patients had increased ALT (> 45 U/L) [17]. The differences between ethnicities and diseases presentation of Q fever might play a role. The most common presentation of acute Q fever was pneumonia in the Netherlands [17] but was hepatitis in Taiwan [14, 16]. The increased transaminase values might reflect disease severity of acute Q fever based on the correlation between CRP, PCT and AST values. However, in a similar study targeting Q fever, scrub typhus, and murine typhus, Chang K [18] et al found no difference in peak transaminase values between patients with and without severe complications. More studies may be warranted to draw a conclusion.

In addition to the positive correlation between the values of CRP, PCT and WBC, their values within 7 days from disease onset were also significantly and positively correlated with the days from doxycycline treatment to defervescence in acute Q fever (Fig. 4). All these results suggested that CRP, PCT and WBC values could be predictors for treatment response of acute Q fever. Overt leukocytosis (WBC > 12,000/uL) was not common in Q fever according to previous studies [14, 17, 18, 21]. The results of using WBC to differentiate disease severity showed inconsistency. Study by de Wit et al found no difference of WBC values between inpatients and outpatients of Q fever [21]. Though both WBC values did not reach the criteria of leukocytosis, Chang K [18] et al found significantly higher WBC values in patients with severe complication than those without in a study of Q fever, scrub typhus, and murine typhus. The WBC counts might reflect disease severity to a certain extent, and our results provided a way to predict the time to defervescence using CRP, PCT and WBC values collectively. The results also corresponded with our previous case report of a patient with acute Q fever granulomatous hepatitis who had a poor response to antibiotics [35], supporting the notion of CRP, PCT and WBC as indicators of treatment response.

Our study has limitations. Firstly, this was a retrospective study and not all patients were obtained CRP and PCT values in both acute and convalescent phases during the treatments. Missing data were complemented by residual specimen, if available. Secondly, this was a single center study. However, since E-Da hospital was in southern Taiwan where acute Q fever, scrub typhus, and murine typhus were endemic, we have included a relatively larger number of clinical cases compared with previous studies in Taiwan. Thirdly, the possible influence of disease severity or underlying diseases could not be evaluated because most of the cases were hospitalized and few had underlying diseases. Fourthly, the different ethnicities and predominant Q fever hepatitis rather than pneumonia in Taiwan might result in difference comparing with other studies.

## Conclusion

Results of this study revealed changes of CRP and PCT values in the acute and convalescent phases of rickettsioses, which might reflect the progression and regression of disease. In febrile patients with markedly high CRP (> 150 mg/L) or PCT values (> 2.0 ng/mL), other infectious etiologies other than rickettsioses alone should be considered. CRP and PCT values, along with clinical symptoms and WBC count, might be helpful in predicting the treatment response to doxycycline in clinical settings.

## Data Availability

The datasets used and analyzed during the current study are available from the corresponding author on reasonable request.

## Abbreviations

ALT: Alanine transaminase
AST: Aspartate transaminase
CRP: C-reactive protein
DNA: Deoxyribonucleic acid
ELISA: Enzyme-linked immunosorbent assay
IFA: Immunofluorescent assay
IgG: Immunoglobulin G
IgM: Immunoglobulin M
PCR: Polymerase chain reaction
PCT: Procalcitonin
Taiwan CDC: Centers for Disease Control, Taiwan
WBC: White blood cell
